# Use of ultrasound biomicroscopy to predict the outcome of anterior segment reconstruction in congenital fibrovascular pupillary membrane with secondary glaucoma

**DOI:** 10.1136/bjo-2022-321762

**Published:** 2022-11-15

**Authors:** Yingting Zhu, Lei Fang, Julius Oatts, Ying Han, Shufen Lin, Liming Chen, Xing Liu, Yimin Zhong

**Affiliations:** 1 State Key Laboratory of Ophthalmology, Zhongshan Ophthalmic Center, Sun Yat-Sen University, Guangdong Provincial Key Laboratory of Ophthalmology and Visual Science, Guangdong Provincial Clinical Research Center for Ocular Diseases, Guangzhou, Guangdong, China; 2 Ophthalmology, University of California San Francisco, San Francisco, California, USA

**Keywords:** Glaucoma, Child health (paediatrics), Imaging, Treatment Surgery

## Abstract

**Aims:**

To evaluate the efficacy and safety of anterior segment reconstruction (ASR) in congenital fibrovascular pupillary membrane-induced secondary glaucoma (CFPMSG) basing ultrasound biomicroscopy (UBM) classification.

**Methods:**

This ambispective cohort study enrolled patients with CFPMSG who underwent ASR between January 2014 and September 2020. Comprehensive ophthalmic examinations and UBM were performed before surgery and postoperatively. The patients were classified into three types according to the UBM configurations. Anterior chamber recovery (ACR) was defined as deepening in anterior chamber (≥1.5 mm all through final follow-up (FFU), while success following ASR was defined as ACR and intraocular pressure (IOP)≤21 mm Hg.

**Results:**

25 eyes of 25 patients underwent ASR (average age at operation 5.8±5.0 months, 48% girls) with FFU 15.8±16.9 months. Enrolled subjects were classified into type Ⅰ (11 eyes), type Ⅱ (11 eyes) and type Ⅲ (3 eyes). After ASR, 23 eyes (92%) achieved ACR, and the mean ACD increased in all groups (p=0.006, <0.001 and 0.003, respectively). Eyes with types Ⅰ and Ⅱ demonstrated a reduction of IOP (p=0.009 and 0.002, respectively). ASR success rate was highest in type Ⅰ (72.9%) compared with types Ⅱ and Ⅲ (18.2% and 0%, respectively; p=0.011). ASR led to decreased number of antiglaucoma medications for type Ⅰ CFPMSG at FFU (p=0.016). No vision-threatening postoperative complications occurred.

**Conclusions:**

ASR for CFPMSG results in increased ACD and improvement in IOP. Postoperative IOP control was best in type Ⅰ CFPMSG but not as effective in types Ⅱ and Ⅲ. UBM-based classification helps to predict the surgical outcome of ASR in CFPMSG.

**What is already known on this topic**

- Congenital fibrovascular pupillary membrane-induced secondary glaucoma (CFPMSG) was likely to be more complicated than CFPM in surgical treatment due to the high intraocular pressure, corneal opacity, shallow or even disappeared anterior chamber and tight adhesion of membrane and lens capsule. There is a lack of cohort study with large series of cases in surgical management for CFPMSG.

**What this study adds**

- We summarised the clinical outcome of anterior segment reconstruction and found that basing ultrasound biomicroscopy (UBM)-based classification, which we first reported previously could be taken as a guide on surgical management and prognosis in this disease.

**How this study might affect research, practice or policy**

- UBM examination and classification should be done for patients with CFPMSG before surgery and in follow-up postoperatively. It is vital for surgical management and prognosis.

## Introduction

Congenital fibrovascular pupillary membrane (CFPM) is a disorder of the anterior segment, which is unilateral and presents with distinctive features including a white pupillary fibrovascular membrane as well as goniodysgenesis.^1–4^ Additionally, this condition has been associated with corneal endothelial decompensation, congenital cataract and abnormalities in the posterior segment. CFPM-induced secondary glaucoma (CFPMSG) occurs when the membrane completely obstructs the pupil, which leads to iris bombé and acute anterior chamber angle closure.^5^ The consequences of this, if left untreated, include permanent vision loss from either deprivational amblyopia (progressive blocking of the pupil) or glaucomatous optic neuropathy from increased intraocular pressure (IOP).^6 7^

Several surgical approaches for removal of the membrane in CFPM have been reported; however, there is limited data on surgical treatment and prognosis for glaucoma secondary to CFPM.^8–10^ Unlike CFPM, surgery for CFPMSG was more complicated due to the high IOP, corneal opacity, shallow anterior chamber depth (ACD) and tight adhesion of membrane and lens capsule. It is more challenging for intraoperative manipulation and postoperative stability.^11 12^ Since paediatric eyes were prone to have more postoperative complications, the goal of initial surgery in this condition is to reform the anterior chamber, ideally improve IOP and establish acceptable visual axis. The purpose of this study was to describe anterior segment reconstruction (ASR) for CFPMSG, including the outcome of the anterior chamber recovery (ACR) and IOP control after ASR, and determine the association between preoperative ultrasound biomicroscopy (UBM) classification with the postoperative clinical outcomes following ASR.

## Methods

### Patients

Consecutive patients of CFPMSG undergoing ASR at Zhongshan Ophthalmic Center, Sun Yat-sen University between January 2014 and September 2020 were included.

Inclusion criteria were as follows: (1) age<16 years at the time of surgery, (2) a diagnosis of CFPM based on previous studies,^13^ (3) IOP>21 mm Hg by repeated measurements, with pupil membrane obstruction, iris bombé and enlargement of cornea and axial length (AL) following definition of paediatric glaucoma by Childhood Glaucoma Research Network.^14^ Patients were excluded if they had bilateral disease or other types of glaucoma, or if they had systemic disease deemed not suitable for general anaesthesia. Demographic data were collected including sex, age at onset of glaucoma, age at operation and eye laterality. After surgery, we checked the patients once a week in the first month, then once a month in the next 2 months, afterwards every 3 months in the subsequent follow-up. Final follow-up (FFU) was defined as the last follow-up visit available for patients who did not require additional surgery and the day before additional glaucoma surgery for those who required it.

### Ophthalmic examination

All patients underwent a comprehensive ophthalmic examination under general anaesthesia. Hand-held slit-lamp biomicroscopy (Keeler, Bucks, England) and slit-lamp photography (BX900; Haag-Streit AG, Koniz, Switzerland) were performed. We classified the corneal opacity of CFPMSG patients as mild (iris details clearly visible), moderate (iris details partially visible) or severe (no iris details visible). IOP was measured by rebound tonometry (Icare PRO; Icare Finland Oy, Helsinki, Finland). Horizontal corneal diameter was measured using callipers. Anterior chamber configuration was assessed with UBM (50 MHz resolution, model SW-3200L; Tianjin Suowei Electronic Technology Co, Tianjin, China). AL and posterior segment evaluation were performed using A-scan and B-scan ultrasonography (Quantel Medical, CF, France; Nidek US-1800, Japan).

### Ultrasound biomicroscopy (UBM)

UBM examination was carried out as previously reported.^13^ Briefly: (1) patients were evaluated under general anaesthesia in the supine position, (2) balanced salt solution was used as the coupling agent maintaining the probe perpendicular to the ocular surface, (3) UBM images were obtained using axial and radial scans at 3, 6, 9 and 12 o’clock positions under standard room illumination, (4) highest quality images from each clock hour and from the central anterior chamber were selected to extract anterior chamber parameters and estimate pupil position. ACD in UBM image was measured as the perpendicular distance from the corneal endothelium to the anterior lens surface. Before or after the surgery, if the anterior lens surface was covered by the pupillary membrane, the perpendicular distance between the corneal endothelium and anterior surface of the iris or CFPM, which was defined as corneal endothelium–anterior surface of the iris or CFPM distance (CAD) was measured. All UBM examinations were carried out by a single experienced examiner (LC).

**Figure 1:**
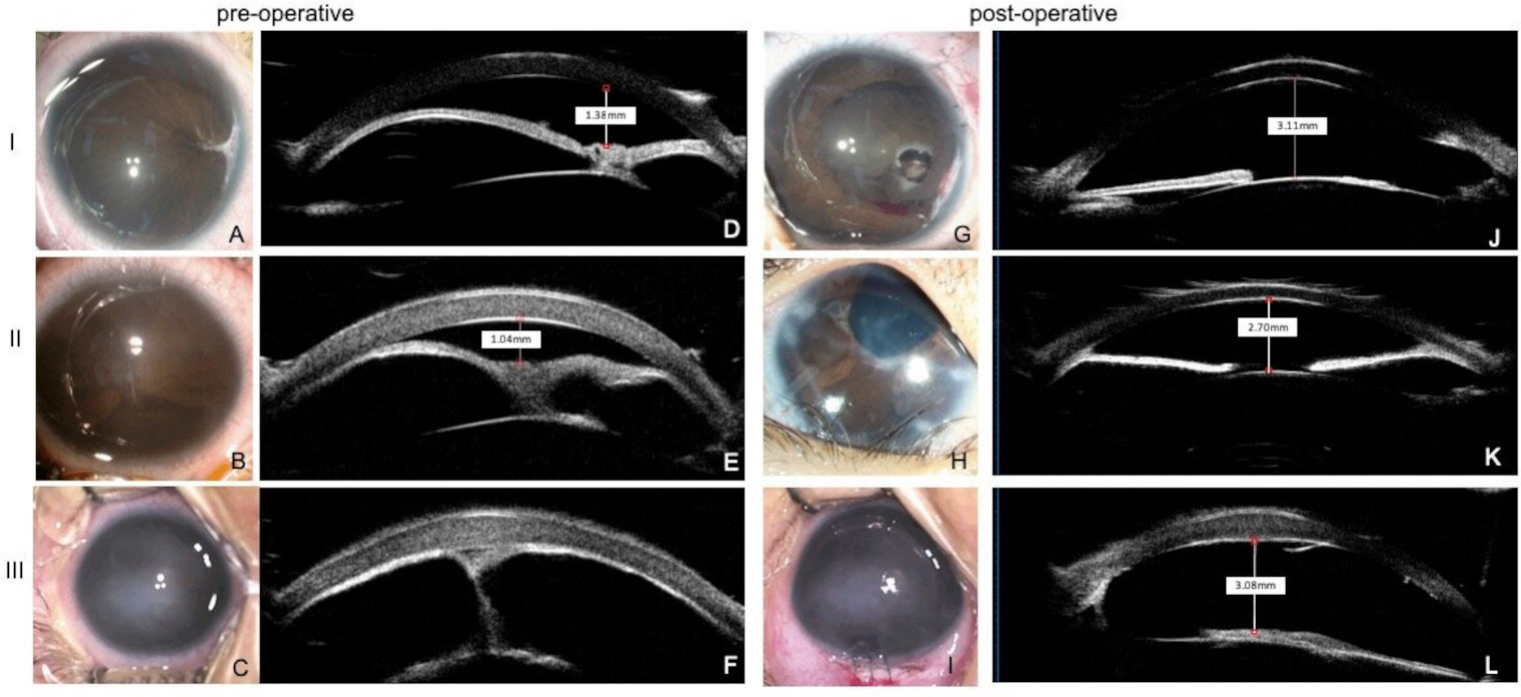
Representative anterior segment photographs and ultrasound biomicroscopy (UBM) images of the three types of congenital fibrovascular pupillary membrane-induced secondary glaucoma (CFPMSG) before (A–F) and after (G–L) anterior segment reconstruction (ASR). Top row: type I CFPMSG (‘U’ shape): 5-month-old girl who presented with mild corneal oedema, complete pupillary obstruction with membrane and iris bombé in her left eye. Preoperative intraocular pressure (IOP) was 21 mm Hg and corneal endothelium–anterior surface of the iris or CFPM distance (CAD) was 1.38 mm. Eight months after ASR, IOP reduced to 15.8 mm Hg and anterior chamber depth (ACD) was 3.11 mm. There was a tight adhesion between the remnant of the membrane and the lens capsule, but the rest of the lens stayed clear. Middle row: type II CFPMSG (‘Y’ shape): 3-month-old boy who presented with moderate corneal oedema, iris stretched to the fibrotic membrane and more severe iris bombé with anterior synechiae in his right eye. Preoperative IOP was 37.2 mm Hg and CAD was 1.04 mm. Thirty months after ASR, IOP was reduced to 22 mm Hg and ACD was 2.7 mm, but the anterior chamber angle remained closed. Bottom row: type III CFPMSG (‘I’ shape or no anterior chamber): 8-month-old girl who presented with severe corneal oedema and complete iris–cornea contact in her left eye. Preoperative IOP was 31.3 mm Hg with CAD of 0 mm. Twelve months after ASR, IOP decreased to 28 mm Hg and the anterior chamber was deep. A,B,E,F,I,J: preoperative; C,D,G,H,K,L: postoperative; A,C,E,G,I,K: anterior segment photography; B,D,F,H,J,L: UBM image.

Iris configuration was recorded and classified as previously described.^13^ Representative images are shown in figure 1. Type Ⅰ (‘U’ shape) was defined as complete obstructed with resultant iris bombé and iridocorneal contact. A thick and hyper-reflective membrane blocks the pupil and is completely adherent to the anterior lens surface. Type Ⅱ (‘Y’ shape) was defined as similar to type Ⅰ but with more severe iris bombé and fibrotic adhesions between the elevated portions of the iris above the invisible pupil. The thick hyper-reflective membrane in this type is larger with iris–lens adhesion. Type Ⅲ (‘I’ shape or no anterior chamber) was defined as complete absence of anterior chamber due to total iridocorneal apposition. Some strands of iris can be seen connected to the membrane on the anterior surface of the lens.

### Anterior segment reconstruction

All surgical procedures were performed by a single surgeon (XL). After patients were placed under general anaesthesia, a planned staged ASR was performed. First, a fornix-based superotemporal conjunctival peritomy is created in the quadrant closest to the occluded pupil. A biplanar tunnelled incision is fashioned at the corneoscleral junction. Before entering the anterior chamber, a clear corneal paracentesis is made opposite the scleral tunnel. A peripheral iridectomy (PI) is then created using tying forceps and iridectomy scissors in the main tunnelled incision. The viscoelastic is used to fill the anterior chamber and synechiolysis of the anterior synechiae is performed using the viscoelastic cannula or sinskey hook. Similarly, the viscoelastic is injected into the posterior chamber through the orifice created by PI, and separation of the adhesions between membrane and anterior lens capsule was performed using blunt dissection. Care is taken to avoid damage to the lens. To allow an alternative clear visual axis, optical iridectomy and pupilloplasty are performed using iridectomy scissors in cases where blunt separation is not successful. Haemostasis is achieved by pressurising the anterior chamber with viscoelastic if needed. The limbal incision is closed with single interrupted 10-0 nylon sutures and the conjunctiva is closed with 8-0 absorbable sutures. IOP was adjusted to physiological level with some of the viscoelastic remained in the eye. Postoperatively, combination drops of dexamethasone and tobramycin are used four times a day, tapered weekly and discontinued after 4 weeks. Atropine 1% ointment is also prescribed daily for 1 week in case of iris synechia postoperatively.

### Success criteria

ASR was considered successful based on postoperative ACD and IOP. Success was defined as (1) ACR, which was defined as improvement in ACD (postoperative ACD/CAD≥1.5 mm) and stablisation without iris bombé until FFU, (2) IOP was ≤21 mm Hg without additional glaucoma surgery and (3) no development of severe vision-threatening complications including corneal decompensation, severe hyphema, choroidal detachment, endophthalmitis or retinal detachment. Failure was defined as ACD/CAD<1.5 mm, or IOP>21 mm Hg with maximum medications and the requirement of an additional operation to lower IOP, or the occurrence of severe complications, such as corneal decompensation, choroidal detachment, severe hyphema or endophthalmitis.

### Statistical analysis

Statistical analysis was performed using SPSS (V.22.0). Descriptive statistics were presented as means with SD, medians with ranges or numbers and percentages as appropriate. For continuous variables, the normality of the distribution was examined using the Kolmogorov-Smirnov test. For comparison between preoperative and postoperative parameters, Student’s paired t-test or Kruskal-Wallis H test were used as appropriate. Analysis of variance, Pearson χ^2^ test and Fisher exact test were used for comparison across UBM-defined subtypes. Bonferroni correction was used for multiple comparisons. The cumulative survival rate was calculated using a Kaplan-Meier survival estimate. Statistical significance was set at p<0.05.

## Results

### Baseline characteristics

Overall, 25 eyes of 25 patients were included in the study, including 13 boys and 12 girls. Demographic and clinical characteristics are summarised in table 1. The average age at onset was 2.5±5.1 months. All cases were unilateral with 15 occurring in right eyes and 10 in left eyes. There was no known family history of pupillary abnormalities or glaucoma in any patient. Based on UBM, 11 eyes (44%) were classified as type Ⅰ, 11 eyes (44%) as type Ⅱ and 3 eyes (12%) as type Ⅲ. All patients underwent ASR as the first surgical treatment at an average age of 5.8±5.0 months. Mean FFU was 15.8±16.9 months (range 2–45 months, type Ⅰ 20.5±13.4 months, type Ⅱ 15.3±22.2 months, type Ⅲ 6.3±4.9 months; p=0.453).

**Table 1:** Ultrasound biomicroscopic classification, demographic and clinical characteristics

No.	UBM type	Sex	Eye laterality	Age at onset (months)	Age at operation (months)	FFU duration (months)	Time prior to secondary glaucoma surgery (months)
1	Ⅰ	M	OD	0.03	4	45	–
2	Ⅰ	F	OS	0.03	4	21	–
3	Ⅰ	F	OD	24	24	15	–
4	Ⅰ	M	OD	0.03	1	12	–
5	Ⅰ	M	OD	0.03	1.5	4	4
6	Ⅰ	F	OD	8	10	37	37
7	Ⅰ	M	OS	7.5	13.5	36	–
8	Ⅰ	F	OS	0.03	2	12	–
9	Ⅰ	M	OD	0	1	15	–
10	Ⅰ	M	OS	0	3.5	23	–
11	Ⅰ	F	OS	0	2	9	9
12	Ⅱ	M	OD	6	9	36	–
13	Ⅱ	F	OD	3	4	72	–
14	Ⅱ	M	OD	3	3	30	30
15	Ⅱ	M	OD	0.03	8	4	4
16	Ⅱ	F	OS	2.7	4.7	7	7
17	Ⅱ	M	OD	0.5	2.5	2	2
18	Ⅱ	F	OD	0.03	7	4	4
19	Ⅱ	M	OS	0.03	8	3.5	3.5
20	Ⅱ	F	OD	0.03	4	4	4
21	Ⅱ	M	OD	0	7	4	4
22	Ⅱ	M	OD	0	4	2	2
23	Ⅲ	F	OS	1	8	12	12
24	Ⅲ	F	OS	5	8	4	4
25	Ⅲ	F	OS	1.5	2	3	3

F, female; FFU, final follow-up; M, male; OD, right eye; OS, left eye; UBM, ultrasound biomicroscopy.

Preoperative CAD was greater in types Ⅰ and Ⅱ eyes compared with type Ⅲ (p=0.002 and 0.011, respectively). Additionally, preoperatively, types Ⅱ and Ⅲ eyes had significantly higher IOP than type Ⅰ (p=0.004 and 0.038, respectively) (table 2).

**Table 2:** Preoperative and postoperative clinical characteristics following anterior segment reconstruction

UBM type	n	IOP (mm Hg, mean±SD)			ACD/CAD (mm, mean±SD)			Ocular hypotensive agents (n, range)		
		preoperative	FFU	P value*	preoperative	FFU	P value*	preoperative	FFU	P value*
Total	25	29.4±5.5	23.2±7.6	**0.002**	1.3±0.9	3.0±0.9	**<0.001**	3 (2-3)	3 (0–3)	0.212
Ⅰ	11	25.8±4.9	18.2±7.2	**0.009**	1.7±0.8	2.8±0.9	**0.006**	3 (2-3)	1 (0–3)	**0.016**
Ⅱ	11	32.1±4.6	26.5±5.7	**0.002**	1.3±0.7	3.1±1.1	**<0.001**	3 (2-3)	3 (0–3)	0.562
Ⅲ	3	32.5±2.9	29.6±4.8	0.421	0	2.7±0.6	**0.003**	3	3	1
P value†		**0.004** ^a^,**0.038** ^b^,0.895^c^	**0.018** ^a^,**0.034** ^b^,1.000^c^		0.264^a^,**0.002** ^b^,**0.011** ^c^	0.802		0.475	**0.012^a^,**0.085^b^,1^c^	

Statistically significant p<0.05 is shown in bold.

*Comparison of IOP and ACD/CAD at FFU with that prior to operation using Student’s paired t-test. Comparison of drugs at FFU with that prior to operation using Mann-Whitney test.

†Comparison of IOP and ACD/CAD among patients with three types of UBM configurations using analysis of variance. Comparison of preoperative drugs and drugs at FFU among patients with three types of UBM configurations using Kruskal-Wallis test. a. type Ⅰ versus type Ⅱ; b. type Ⅰ versus type Ⅲ; c. type Ⅱ versus type Ⅲ.

ACD, anterior chamber depth; CAD, corneal endothelium–anterior surface of the iris or CFPM distance; FFU, final follow-up; IOP, intraocular pressure; UBM, ultrasound biomicroscopy.

### Clinical outcomes

**Figure 2:**
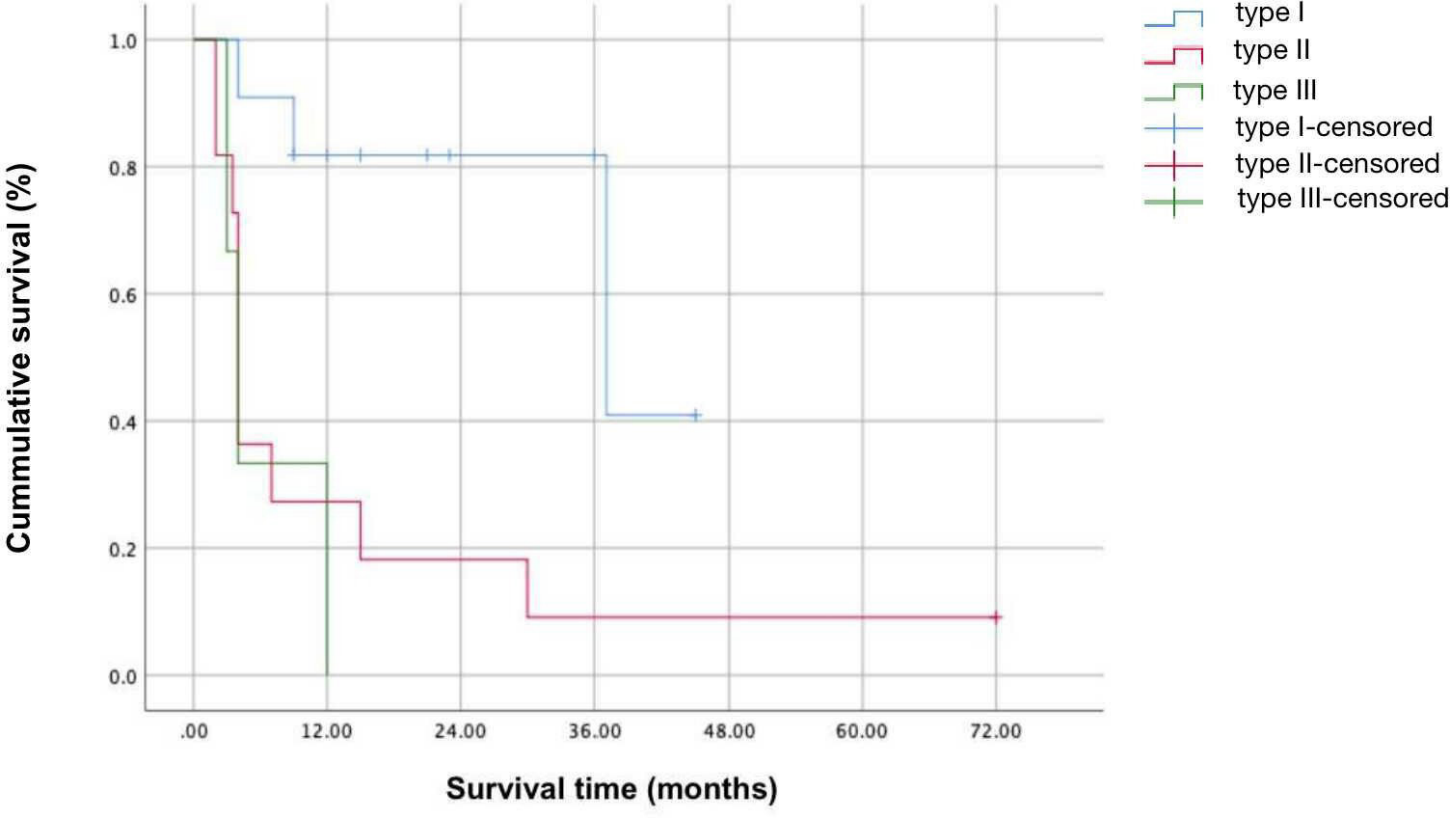
Cumulative survival curves in type Ⅰ (n=11), type Ⅱ (n=11) and type Ⅲ (n=3) eyes with congenital fibrovascular pupillary membrane-induced secondary glaucoma that underwent anterior segment reconstruction.

Following ASR, the anterior chamber deepened significantly in most eyes as shown in figure 1. At FFU, the ACR rate of all eyes was 92% (23/25). Overall mean ACD increased significantly from 1.33±0.88 mm preoperatively to 2.96±0.95 mm postoperatively (p<0.001) (table 2). Total success rate following ASR for all eyes was 40%. The success rate varied by type: 72.7% (type Ⅰ), 18.2% (type Ⅱ) and 0% (type Ⅲ). Overall mean IOP at FFU had improved from 29.4±5.5 mm Hg preoperatively to 23.2±7.6 mm Hg postoperatively (p=0.002) (table 2). Types Ⅱ and Ⅲ eyes had higher IOP at FFU compared with type Ⅰ (p=0.018 and 0.034, respectively). No severe complications were recorded in any eyes. The cumulative success rate calculated by Kaplan-Meier survival analysis is presented in figure 2.

For type Ⅰ eyes, all patients demonstrated significant anterior chamber deepening postoperatively (p=0.006). At FFU, average IOP for this group was significantly lower than preoperatively from 25.8±4.9 mm Hg to 18.2±7.2 mm Hg (p=0.009, table 2). Eight eyes (72.7%) achieved IOP<21 mm Hg (3 with ocular hypotensive medications and 5 without). The remaining 3 eyes (27.3%) had IOP elevation requiring glaucoma drainage device implantation later.

For type Ⅱ eyes, the anterior chamber was significantly deeper than that before ASR (p<0.001) (table 2), with 9 eyes (81.8%) achieved ACR. The numbers 21 and 22 needed additional ASR due to relapse of iris bombé and shallowed anterior chamber. The average IOP at FFU was decreased (p=0.002, table 2). But most type Ⅱ eyes (9/11, 81.8%) had poorly controlled IOP after the first ASR and need additional surgery, including AGV implantation (7 eyes), AGV implantation combined with pupilloplasty (1 eye) and repeated ASR (1 eye).

For type Ⅲ eyes, the anterior chamber was significantly deeper comparing with preoperation (p=0.003) (table 2). But all three eyes showed increased IOP after ASR and needed additional surgery. Two eyes (numbers 23 and 25) received AGV implantation after ASR and one eye (number 24) received diode laser transscleral cyclophotocoagulation.

Before surgery, all patients suffered from severe corneal opacity. At FFU, we found that 8 out of 11 patients in type Ⅰ and 4 out of 11 patients in type Ⅱ could achieve moderate corneal opacity.

The number of postoperative ocular hypotensive medications for all 3 types of CFPMSG patients was 3 (0–3), which was not significantly different compared with preoperatively (3 (2–3), p=0.21). The number of medications for type Ⅰ eyes was significantly less after the first ASR compared with preoperatively (p<0.016). But no significant difference was found in type Ⅱ and Ⅲ. At FFU, the number of medications for type Ⅰ was significantly less than type Ⅱ (type Ⅰ: 1 (0–3), type Ⅱ: 3 (0–3), type Ⅲ: 3; p=0.012 types Ⅰ and Ⅱ).

## Discussion

CFPM is a rare unilateral neurocristopathy presenting as a fibrous membrane covering the pupil, which affects anterior chamber and anterior chamber angle development and can lead to permanent vision loss if not treated.^1 2 4^ Patients with CFPM are at risk to develop complete pupillary closure, which can result in iris bombé and glaucoma.^3 8^ Generally, the secondary glaucoma that develops in CFPM is difficult to control and literature on long-term outcomes is lacking. Liang *et al* published a small case series and nearly 50% of the 9 eyes with CFPMSG had uncontrolled IOP despite surgery.^10^ Additionally, the risk factors related to the surgical outcome of CFPMSG remain unclear. In the current study, we found the classification system of UBM configurations we established for CFPMSG^13^ is associated with the surgical outcome of ASR. ASR is more effective to control IOP for eyes with type Ⅰ CFPMSG than that in type Ⅱ and type Ⅲ eyes.

Sporadic case reports, mainly as part of study in CFPM, have shown the effects of surgical treatment in CFPMSG, where PI and anterior chamber reconstruction were performed for an initial treatment as we did.^3 5 6 8–10^ Several patients relieved with decreased IOP after the operation.^3 5 8 9^ But most glaucoma patients needed multiple surgeries to control IOP during the follow-up, including repeated pupilloplasty, Ahmed valve implantation or other glaucoma surgeries.^3 6 10^ Although ASR is frequently performed in CFPM,^15–17^ as the small number of the previous reports in CFPMSG, the effectiveness and safety of ASR in CFPMSG is unclear. Most importantly, there were no systematic description or classification of UBM in CFPMSG before,^9 18^ so the detailed factors related to the surgical outcomes of ASR in CFPMSG are still unknown.

Our relatively large case series demonstrated that increased severity of the fibrovascular membrane proliferation and iris adhesion defined by UBM are associated with a lower rate of surgical success in CFPMSG. Among patients with the three types of UBM configurations, the type Ⅲ eyes had the shallowest anterior chamber, while the type Ⅱ and type Ⅲ eyes had more extensive synechia and significantly higher IOP than that of type Ⅰ. The type Ⅱ and type Ⅲ eyes may represent the CFPMSG patients referring at a later age with prolonged adhesion of the anterior segment, including the anterior chamber angle. While the type Ⅰ eyes achieved the highest success rate (72.7%), 81.8% of type Ⅱ and all the type Ⅲ eyes needed additional surgery. These results suggest that timely diagnosis and prompt surgical intervention might prevent catastrophic vision loss.^7^

Another important finding from our study is that the ACR rate of all CFPMSG eyes was 92% (23/25). Although additional glaucoma surgery may be needed in some of these patients, deep and stable anterior chamber with improved cornea clarity and decreased IOP after ASR might facilitate further glaucoma filtering surgery or cataract surgery.

During the follow-up, we also found that the lens was still clear in most patients and optical iridectomy could bring relative enough visual axis for CFPMSG. Moreover, cataract surgery in infants with high IOP may cause more significant postoperative inflammation^19 20^ and increased membrane recurrence.^21^ So we advised against lensectomy in the initial surgery in CFPMSG.

As corneal opacity prevented gonioscopy in these patients, UBM was performed after ASR to evaluate the anterior chamber angle as shown in online supplemental table 1. We found only two patients achieving partially or completely open anterior chamber angle after ASR. During follow-up (12–45 months, mean 22.4±12.1), eight children with close angle after ASR (four with glaucoma medications and four without) demonstrated normal IOP (supplementary figure 1). The inner mechanisms of maintenance of normal IOP in the patients without medications are still unknown. Decreased aqueous humour secretion may play a role in these patients. Long-term follow-up is needed.

10.1136/bjo-2022-321762.supp1 Supplementary data

There were several limitations in this study. First, biases were unavoidable as nature of some retrospective data. Second, due to the difficulty for examination of paediatric patients, there were some missing data.

In conclusion, this study indicates that our classification system based on UBM imaging could be used to predict the surgical outcomes of CFPMSG patients undergoing initial ASR. ASR could effectively reduce IOP and restore anterior chamber, especially in type Ⅰ CFPMSG. This study highlights that ASR is feasible for type Ⅰ CFPMSG, and could also provide possibility for type Ⅱ and type Ⅲ patients to receive further glaucoma surgery if needed.

10.1136/bjo-2022-321762.supp2 Supplementary data

## Data Availability

Data are available upon reasonable request.
