# Supplementary material for: Use of ultrasound biomicroscopy to predict the outcome of anterior segment reconstruction in congenital fibrovascular pupillary membrane with secondary glaucoma
Source: Br J Ophthalmol. 2022 Nov 15;108(1):65–70. doi: 10.1136/bjo-2022-321762 (PMC10803978; doi:10.1136/bjo-2022-321762)
Supplement: Supplementary data [file bjo-2022-321762supp001.pdf]

SupplementaryTable 1. Postoperative clinical characteristics and ultrasound biomicroscopy of the CFPMSG patients

No.	Preoperative UBM type	IOP at FFU (mmHg)	Glaucoma drugs at FFU(n)	Postoperative UBM
1	Ⅰ	20.4	1	AC
2	Ⅰ	12.3	0	AC
3	Ⅰ	10.9	0	AC
4	Ⅰ	18.9	0	AC
5	Ⅰ	25.3	3	AC
6	Ⅰ	25.8	3	AC
7	Ⅰ	18.8	2	AC
8	Ⅰ	10	1	AC
9	Ⅰ	10	0	AC
10	Ⅰ	16.6	0	AO
11	Ⅰ	31.4	3	AC
12	Ⅱ	19	3	AC
13	Ⅱ	18	0	1/2 AO
14	Ⅱ	22	3	AC
15	Ⅱ	25.8	3	AC
16	Ⅱ	32.9	3	AC
17	Ⅱ	27	3	AC

18	Ⅱ	32.9	3	AC
19	Ⅱ	30.2	3	AC
20	Ⅱ	29	3	AC
21	Ⅱ	33	3	AC
22	Ⅱ	21.2	3	AC
23	Ⅲ	28	3	AC
24	Ⅲ	25.8	3	AC
25	Ⅲ	35	3	AC

UBM, ultrasound biomicroscopy; IOP, intraocular pressure; FFU, final follow up; AO, angle open; AC, angle closure
