# Supplementary figures and images for: Use of ultrasound biomicroscopy to predict the outcome of anterior segment reconstruction in congenital fibrovascular pupillary membrane with secondary glaucoma
Source: Br J Ophthalmol. 2022 Nov 15;108(1):65–70. doi: 10.1136/bjo-2022-321762 (PMC10803978; doi:10.1136/bjo-2022-321762)

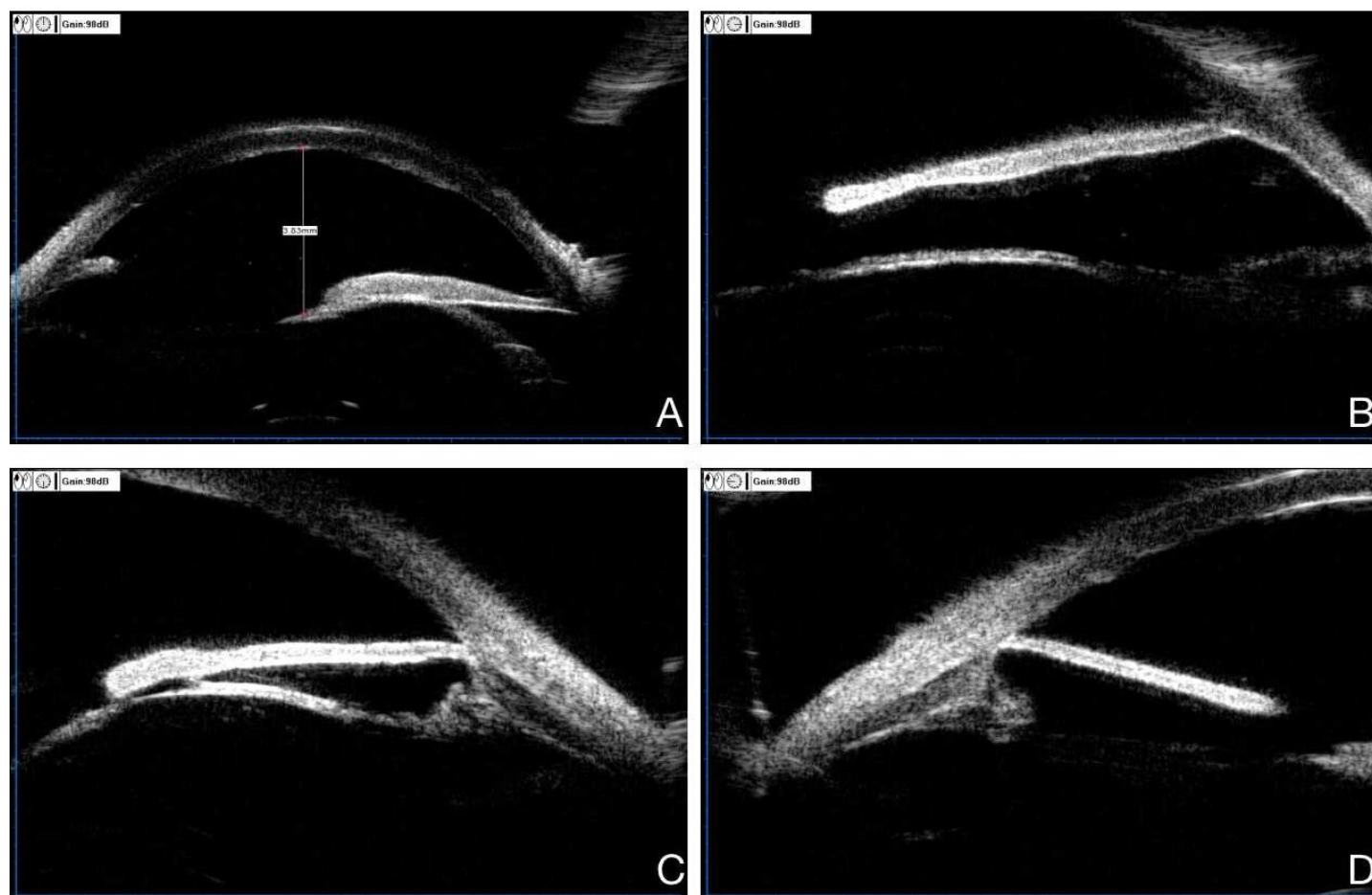

Supplement: Supplementary data [file bjo-2022-321762supp002.pdf]
